# Management of Chinese Rose Beetle (*Adoretus sinicus*) Adults Feeding on Cacao (*Theobroma cacao*) Using Insecticides

**DOI:** 10.3390/insects7020028

**Published:** 2016-06-24

**Authors:** Helen Spafford, Alexander Ching, Megan Manley, Chelsea Hardin, Harry Bittenbender

**Affiliations:** 1 Department of Plant and Environmental Protection Sciences, College of Tropical Agriculture and Human Resources, University of Hawai’i Manoa, 3050 Maile Way, Gilmore Hall 310, Honolulu, HI 96822, USA; aching2@hawaii.edu (A.C.); manleym@hawaii.edu (M.M.); chardin1993@gmail.com (C.H.); 2 Department of Tropical Plant and Soil Sciences, College of Tropical Agriculture and Human Resources, University of Hawai’i Manoa, 3190 Maile Way, St John Hall Room 102, Honolulu, HI 96822, USA; hcbitt@hawaii.edu

**Keywords:** entomopathogen, neem, pest management, ornamental, biopesticide

## Abstract

The Chinese rose beetle (*Adoretus sinicus* Burmeister (Coleoptera: Scarabaeidae)) is an introduced, widely-established pest in Hawai’i. The adult beetles feed on the leaves of cacao (*Theobroma cacao* L.), which can lead to defoliation and even death of young trees. We evaluated the impact of five commercially available products with different active ingredients (imidacloprid, azadirachtin, *Beauveria bassiana (*Bals.-Criv.) Vuill., kaolin clay, and pyrethrin) and the presence or absence of weed mat cover in reducing adult beetle feeding on sapling cacao in the field. The use of weed mat cover reduced feeding damage compared to the untreated control, as did foliar application of imidacloprid, azadirachtin, and *B. bassiana*. In the laboratory, field-collected adult beetles were presented cacao leaf samples dipped in one of the five products and compared to a control. Beetles exposed to pyrethrin died rapidly. Among the other treatments, only exposure to imidacloprid significantly reduced survival relative to the control. Beetles fed very little on leaf samples with azadirachtin but their longevity was not significantly reduced. Imidacloprid, azadirachtin, and weed mat application had the most promise for reducing adult Chinese rose beetle feeding damage in young cacao and deserve further investigation for successful management of this significant pest.

## 1. Introduction

The Chinese rose beetle (CRB) (*Adoretus sinicus* Burmeister) (Coleoptera: Scarabaeidae), a scarab beetle native to Japan and Taiwan, is known to be an economically important pest in Southeast Asia, China, Indonesia, Cambodia, Singapore, Thailand, Vietnam, the Mariana Islands, the Caroline Islands, and many other Pacific Islands [1,2]. The CRB was first found on O’ahu, Hawai’i in 1891 and has since established itself as a significant plant pest on all major Hawai’ian islands [3]. The larvae of CRBs are subterranean and feed on underground decomposing plant matter [3,4]. After pupation in the soil, the adults emerge. Adult CRBs are nocturnal and feed on the leaves of more than 500 plant species, including cacao (*Theobroma cacao* L. (Malvaceae) [1,3,5]. The shot-hole feeding and skeletonization of the young cacao leaves by CRBs can lead to reduced growth, defoliation, and even death of young trees, thus creating problems for orchard establishment [1].

Cacao production supports a multi-billion dollar chocolate industry, and US domestic production of cacao is currently limited to Hawai’i [6]. Commercial production of cacao in Hawai’i began the early 1900s [7] and experienced fluctuating success until the Dole Food Company planted 20 acres in the late 1990s [8]. Dole Food Company remains the largest grower with approximately 22 acres planted in cacao, and there are a number of other smaller growers located throughout the islands [6,9]. The high value of Hawai’i -grown cacao (estimated at 2–4 times higher than other cacao traded in world markets [8]) has spurred the development of new cacao orchards on other Hawai’ian islands. However, destructive CRB feeding has the potential to limit the profitability of these investments.

Many methods of control for CRBs in Hawai’i have been investigated [1]. Immature stages have been affected by classical biological control agents, including parasitoids and pathogens [1], and both immature and adult stages are vulnerable to natural biological control from vertebrates such as chickens, cane toads, and geckos [6]. Nighttime illumination has been shown to reduce adult CRB feeding in small cacao fields [2]. However, many of these approaches are not practical or effective for commercial cacao production, and growers still require insecticides. Worldwide, agrochemicals are frequently used for management of cacao pests [10]. In this study, we evaluated five commercially available pest management products with the active ingredients azadirachtin, pyrethrin, imidacloprid, kaolin clay, and *Beauveria bassiana* (Bals.-Criv.) Vuill. for their effectiveness in reducing feeding damage from adult beetles in a young cacao orchard, and the impact of the same products on the mortality of adult beetles in the laboratory. In Hawai’i, few products are approved for use in cacao for management of CRBs, but there are a number of other products that may have efficacy against CRBs [1]. Although there has been evaluation of antifeedent properties of azadirachtin against CRBs on strawberry and bean, there are no published results of the effectiveness of azadirachtin or pyrethrin on CRBs in cacao crops [11]. Imidacloprid is effective on CRBs [1], but has not been tested in cacao. Kaolin clay has been shown to reduce feeding of foliar feeding insects, including beetles in apples [12]. *Beauveria bassiana* is being used on coffee as a biological control for the coffee berry borer. In our study, it was not expected that *B. bassiana* would have an effect on adult beetle mortality or reduce feeding damage, but we included it in the experiments to evaluate the potential short-term value of this product. The field study also evaluates the effect of weed mat cover on adult beetle feeding damage.

## 2. Materials and Methods

### 2.1. Feeding Damage in the Field

**Figure 1:**
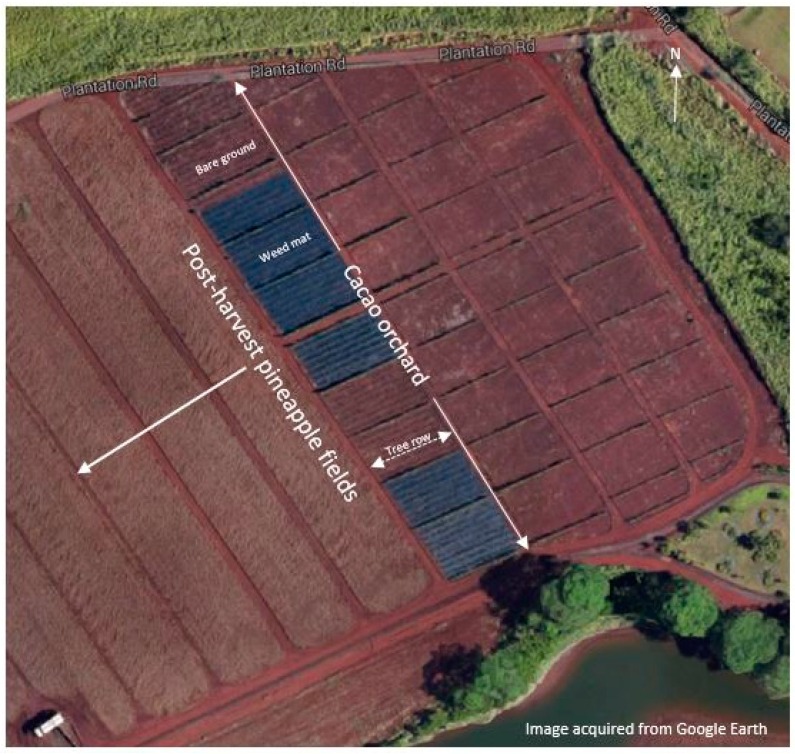
Map of cacao field at Dole Plantation, Helemano, O’ahu, HI, USA.

The field study was conducted in a 0.8 ha cacao orchard at the Dole Plantation, Helemano, O’ahu, Hawai’i (Figure 1). The field had previously been planted with pineapple. It was bordered on the north by weedy grasses, on the south by a small river and on the east and west by a post-harvest pineapple field.

After initial germination and growth in a greenhouse, cacao plants (seedlings of mixed *forastero*, *criollo*, and *trinitario* varieties) had been transplanted to the field at various times over several years. The trees in the orchard were planted in rows in a NE-SW direction with approximately 25–30 trees in each row (Figure 1). The youngest trees were planted at the north-western end of the orchard and the oldest were at the south-eastern end (Figure 1). If a tree within the orchard died it was replaced with a younger tree. Thus, the orchard consisted of trees of mixed ages between 6–24 months old.

Before the study, CRB damage in the orchard had been high and a number of trees had died from persistent defoliation. Several approaches had been attempted to protect the trees. The youngest trees were protected from wind and beetles by a circular wire cage covered with plastic sheeting, but once the cages were removed the trees were attacked by CRBs. The trees had also been previously treated with foliar application of carbaryl (Sevin®, Bayer CropScience LP, Research Triangle Park, NC, USA), which provided a single night of protection from the beetles. While effective for a very short period, carbaryl had to be re-applied on a daily basis to provide protection if beetle activity was high. Weed mat (Sunbelt, DeWitt Woven Ground Cover Landscape Fabric, Sikeston, MO, USA) had been placed in sections of the orchard to reduce adult emergence from within the orchard (Figure 1). However, it was believed that an adjacent post-harvest pineapple field was the primary source of CRBs, and this post-harvest residue was treated with diazinon (Syngenta Crop Protection Canada, Guelph, ON, Canada) to reduce adult beetle emergence. The orchard managers also had placed solar powered lights in the field following the recommendation of McQuate and Jameson [2]. However, all the lights were stolen a few hours after placement. Despite these efforts, the level of damage from CRBs remained high.

Two areas within the field were selected for the experiment (each one a single block). Within each area a portion of the ground was covered with weed mat, and the rest remained uncovered (bare). The weed mat had been placed by the orchard managers some time before the experiment and the selection of the experimental area was made on the basis of having both bare and covered portions. Individual cage-protected trees in each area were visually inspected and selected for use in the experiment on the basis of overall plant health and little or no CRB damage on young newly expanded leaves. Each selected tree (replicate) was marked with flagging tape and three completely undamaged leaves (sub-samples) on each tree were also flagged. An equal number of trees were selected from each of the two blocks (areas) with five trees in each covered and uncovered area selected for each treatment (n = 140). Each flagged tree was assigned randomly to one of seven treatments (Table 1) before product application and this assignment was identified by a unique color of flagging tape. In order to evaluate the impact of proximity to the post-harvest field, we measured the distance of each selected tree from the field edge bordering the post-harvest pineapple field.

On 3 October 2014, upon entering the field for treatment, any remaining protective cages on the trees selected for treatment were removed from the trees. All treatments were applied to flagged trees using either a hand-held sprayer containing the diluted product or water for foliar applications sprayed to drip (Table 1). The soil drench was applied around the base of each tree by pouring 0.24 L of drench solution (0.029 mL active ingredient per tree) without regard to stem diameter, equivalent to the label rate. (Table 1). Before product application the flagged leaves were re-inspected for adult feeding damage. If damage was observed and a suitable substitute leaf on the plant could not be found, the replicate was discarded (n = 8). Each tree that remained in the experimental group was sprayed with the assigned treatment until all foliage was wet.

Three days following treatment, on 6 October 2014, each flagged leaf on each tree was visually inspected for adult feeding damage and scored according to the leaf area consumed (Table 2). The scale was developed before the field scoring by collecting leaves from a nearby cacao orchard in a manner similar to Smith et al. [6].

Once the field assessment was completed, the median damage scores were analyzed in an ordinal logistic regression model (logit link) using Minitab 16 (2013 version 16.2.4 for Windows, Minitab Inc., State College, PA, USA) to compare the effect of product treatments and weed mat cover on leaf damage with distance from the post-harvest pineapple field included as a covariate. The block was included as a factor.

### 2.2. Adult Beetle Mortality in the Laboratory

To test for the direct effects on beetle mortality we conducted a laboratory bioassay wherein cacao leaves were dipped in either one of the five products or water (control) and then fed to CRB adults. The cacao leaves used in the experiment and fed to the beetles came from established cacao trees grown on the University of Hawai’i, Manoa campus and had not been previously sprayed with any insecticide. Leaf samples (16 cm^2^) were cut from the edge of the leaves towards the center vein using scissors (sanitized in isopropyl alcohol), were then dipped in a treatment, and suspended with small clothespins on a wire drying rack until dry. Once fully dried, the samples were placed individually in a Petri dish lined with filter paper. If the beetles consumed the entire sample, the sample was replaced by another one that had been similarly treated.

Adult CRBs were collected from home and community gardens in Manoa Valley, O’ahu, Hawai’i, HI, USA. These populations of beetles had not been exposed to any insecticides within any of these sites. In the laboratory, beetles were placed collectively in a cage with harvested cacao leaves in order to habituate them to feeding on cacao and to acclimate them to the laboratory environment. After a minimum of three days, actively crawling beetles were placed individually in plastic Petri dishes (10 cm diameter) lined with moistened (0.3 mL water) filter paper (9 cm diameter, Lab Nerd, Avogadros Lab Supply, Inc., Shamong, NJ, USA) and a cacao leaf sample (4 cm × 4 cm square; 16 cm^2^) that had been dipped in either one of five commercially available products or water (control) (Table 3). Each Petri dish was sealed with parafilm (Parafilm ‘M’ Laboratory Film, Neenah, WI, USA) and placed in an incubator (21 °C; 12:12 h light:dark). Beetles were checked daily for mortality. Observations of feeding on the leaf samples were made.

The mean longevity of adult beetles was determined by the number of days from when the beetle was placed in the Petri dish until the time of death. The effect of product treatment or water on longevity of adult beetles was analyzed using a one-way ANOVA using Minitab 16 (2013 version 16.2.4 for Windows, Minitab, Inc., State College, PA, USA) and comparison of means was conducted using a Tukey test. Because the impact of pyrethrin on mortality of beetles resulted in no survival beyond one day, these data were removed from the statistical analysis. The data met the assumptions of normality and equal variance and were analyzed un-transformed.

## 3. Results

### 3.1. Feeding Damage in the Field

Feeding damage from CRBs on the treated trees ranged from none to greater than 80 percent of leaf area consumed. The presence of weed mat cover reduced the median feeding damage rating by 50% from 4 to 2 (Χ^2^ = 6.9, df = 1, *p*-value = 0.009). The damage rating was also affected by product application (Table 4; Χ^2^ = 27.8, df = 6, *p*-value < 0.001). Trees treated with the azadirachtin, foliar application of imidacloprid, pyrethrin, and *B. bassiana* had lower median damage ratings than the trees sprayed with water (control) (*p*-value < 0.05) (Table 4). Damage ratings for trees given the soil application of imidacloprid or foliar application of kaolin clay were not different than those sprayed with water (control) (*p*-value < 0.05) (Table 4). The feeding damage rating was unrelated to the distance of the tree from the post-harvest pineapple field (*p*-value = 0.14).

### 3.2. Adult Beetle Mortality in the Laboratory

Adult CRBs died quickly when exposed to pyrethrin. No specimen exposed to the pyrethrin survived for more than a day, and it was observed that many died within hours of placement in the Petri dish (Table 5). The leaves treated with pyrethrin had no feeding damage.

Longevity of adult CRBs differed in the other five treatments (Table 5; *F*_4,52_ = 4.3, *p*-value = 0.004). Beetles in the control treatment lived an average of 19 days, but as long as 32 days, and actively fed on the leaf samples. An entire sample was consumed in as little as five days, and thus was often replaced several times during the experimental period. The beetles placed in Petri dishes with leaf samples treated in imidacloprid did not live as long as those in the control treatment (Table 5). Very little leaf material was consumed by beetles exposed to imidaloprid and some did not eat at all. Almost no leaf material was consumed by beetles in the azadirachtin treatment; only one beetle in this group consumed any leaf material. Although longevity of beetles in the azadirachtin was less than that of the beetles in the control treatment, it was not statistically significant (Table 5). All the beetles in the kaolin clay treatment did feed on the leaves, but only about 50% of leaf area was consumed and longevity was also not statistically different than the control (Table 5). The beetles in the *B. bassiana* treatment consumed the leaf samples in the same manner as the water-treated control and did not experience any greater mortality than those in the control treatment (Table 5).

## 4. Discussion

Without any form of protection, young cacao leaves are vulnerable to adult beetle CRBs feeding. In this study, previously undamaged cacao leaves encountered a 60% reduction in leaf area on average within just a few days of exposure to beetles; some were completely skeletonized. We evaluated several commercially available products for their effectiveness in reducing adult CRBs feeding and longevity. Foliar applications of imidicloprid, azadirachtin, pyrethrin, and *B. bassiana* were the most effective at protecting leaves on young cacao plants. Among those products effective in the field, only pyrethrin and imidicloprid were also effective in significantly reducing adult longevity in the laboratory.

Imidacloprid is used for the control of sucking insects, including rice hoppers, aphids, thrips, whiteflies, termites, turf insects, soil insects, and some beetles, by interfering with the function of the insect nervous system. Although broadly effective, imidacloprid is of concern for its non-target impacts, particularly in honeybees, and has been implicated in colony-collapse disorder [13,14] yet more recent reports exonerate imidacloprid; however, bees are not involved in pollination nor appear to visit cacao flowers. As a systemic insecticide applied as a soil drench imidacloprid was ineffective in reducing CRB damage. It may be that the time taken for the plant to absorb and translocate the insecticide was too long relative to the rapidity with which the beetles found the young cacao leaves. The rate of imidacloprid absorption applied as a soil drench may have also been influenced by heavy rains immediately preceding the application of treatments. For example, reduced watering prior to application of imidacloprid to poinsettia by sub-irrigation has been shown to increase the rate of absorption of imidacloprid and soil drench application has relatively slow rates of absorption [15]. Had the trees been provided with continued cover for a brief period after soil drench application the level of CRB damage might have been reduced.

Azadirachtin is used to control or repel insect pests such as whiteflies, leafminers, fungus gnats, thrips, aphids, and many leaf-feeding caterpillars. The active ingredient of azadirachtin, nortriterpenoid, exhibits antifeedant, reproductive, and insect growth regulator effects on the immature stages of insects [16]. In the laboratory, adult CRBs exposed to azadirachtin treated leaves did not die faster than those beetles in the control treatment; however, it was our observation that they ate less than beetles in the control treatment, suggesting that azadirachtin may have had a repellent effect. This hypothesis is consistent with the field study where feeding damage was lower on azadirachtin treated trees. However, we did not systematically quantify the leaf area consumed, and our results are suggestive of repellent or anti-feedent impacts on CRBs rather than acute toxicity as was described by Tsutsumi et al. [11].

*Beauveria bassiana* is a fungus that is used as a biocontrol agent for insect pests such as whiteflies, aphids, thrips, psyllids, mealybugs, weevils, leafhoppers, mites, caterpillars, fungus gnats, shoreflies, coffee berry borers, and leaf-feeding insects [17]. The fungus causes disease inside insects when hyphae grow directly through the cuticle to the inner body where it proliferates and produces toxins, draining the insect of nutrients [17]. In the field experiment, the foliar application of *B. bassiana* reduced feeding damage relative to the control. However, given that it is unlikely to cause immediate mortality (as shown in the laboratory experiment), the reduction in damage may be due to other properties of the mixture, such as the adjuvant or repellent properties of either *B. bassiana* or other components of the mixture. In the field experiment, the beetles had the option to move to another plant, but the laboratory assays were no-choice and the beetles did not have the option to disperse. The beetles that ate the leaves dipped in *B. bassiana* did not have greater mortality.

In spite of pyrethrin’s relatively moderate performance in the field, in terms of immediate mortality, it was extremely effective at killing adult CRBs in the laboratory. Exposure to light, high humidity levels or wind may have reduced the effectiveness of this product in the field when it might otherwise be a very effective insecticide for CRB control [18].

The kaolin clay treatment was not very effective at reducing feeding damage or longevity. The average damage rating in the field was >40%, and in the laboratory experiment the beetles also consumed about 50% of the leaf material. Application of kaolin clay left a powdery white coating on treated leaves. The kaolin clay coating may have had a minor impact on reducing CRB feeding, but the laboratory assay confirmed that it was not toxic. As a measure to protect young cacao trees from CRB damage, kaolin clay appears to be ineffective.

We also evaluated the impact of weed mat cover on adult feeding damage. The presence of these mats reduced the levels of damage, but the mechanism is unclear. The mats were placed under the assumption that they would restrict emergence of adults from the soil. We did notice many emergence holes in the uncovered rows of cacao, and it may be that there were many adult beetles emerging in the few days between treatment and evaluation of leaf damage. However, if we assume the majority of adults active during the experimental period were already present in the field and surrounding area and were not newly emerged, then the question of why the weed mats reduced adult feeding damage remains unanswered.

## 5. Conclusions

Imidacloprid, azadirachtin, and possibly *B. bassiana* and pyrethrin show promise for management of adult CRB feeding damage in young cacao. The protection afforded by these products may enable the trees to grow sufficiently. Future research should focus on azadirachtin, its residual activity, and/or whether it leads to direct mortality and is repellent in the field. *Beauveria bassiana* also may be an effective protectant; however, further research needs to be conducted to evaluate the mechanism by which *B. bassiana* led to reduced damage in the field. Given that our study only evaluated the effectiveness of these products for a short period after application, further studies should be conducted to evaluate the long-term effectiveness of these products. The mechanism underlying the effectiveness of weed mats also should be determined, because this appeared to be also effective in reducing adult CRB feeding.

## Figures and Tables

**Table 1:** Treatments including active ingredients, product name, product source, method of application, and dilution applied for management of Chinese rose beetle on young cacao in the field.

Active Ingredient/Treatment	Product Name	Source	Method of Application	Dilution
Azadirachtin	Azatrol® EPA Reg. No. 2217-836	Gordon’s Professional Turf and Ornamental Products, PBI-Gordon Corporation Kansas City, MO, USA	Foliar spray	15.8 mL/1 L water
*B. bassiana*	BotaniGard® EPA Reg. No. 82074-2	Laverlam International Corp., Butte, MT, USA	Foliar spray	0.4 mL/1 L water + 0.21 mL Silwet L-77 Ag spray adjuvant (spreading surfactant)
Imidacloprid	Admire® Pro^TM^ EPA Reg. No. 264-827	Bayer CropSience LP Research Triangle Park, NC, USA	Soil drench	0.29 mL/1 L water
Imidacloprid	Admire® Pro^TM^ EPA Reg. No. 264-827	Bayer CropSience LP Research Triangle Park, NC, USA	Foliar spray	0.04 mL/1 L water
Kaolin clay	Surround® WP Crop Protectant EPA Reg. No. 61842-18	NovaSource, Tessenderlo Kerley, Inc. Phoenix, AZ, USA	Foliar spray	60 g/1 L water + 21.1 mL Attach® adjuvant (spreader-sticker)
Pyrethrin	Prentox Pyronyl^TM^ Crop Spray EPA Reg. No. 655-489	Prentiss Incorporated LLC, Sandersville, GA, USA	Foliar spray	2.64 mL/1 L water
Water (control)			Foliar spray	

**Table 2:** Grading scale to estimate percent feeding damage of Chinese rose beetle on cacao leaves.

Grade	Percent Feeding Damage
0	0
1	1–9
2	10–19
3	20–29
4	30–39
5	40–59
6	60–79
7	80–99

**Table 3:** Treatments, including active ingredients, product name, product source and dilution, applied for management of Chinese rose beetle on cacao leaf samples in the laboratory.

Active Ingredient/Treatment	Product Name	Source	Dilution
Azadirachtin	Azatrol® EPA Reg. No. 2217-836	Gordon’s Professional Turf and Ornamental Products, PBI-Gordon Coorporation Kansas City, MO, USA	15.8 mL/1 L water
*B. bassiana*	BotaniGard® 22WP EPA Reg. No. 82074-2	Laverlam International Corp., Butte, MT, USA	0.41 mL/1 L water
Imidacloprid	Admire® Pro^TM^ EPA Reg. No. 264-827	Bayer CropSience LP Research Triangle Park, NC, USA	0.04 mL/1 L water
Kaolin clay	Surround® WP Crop Protectant EPA Reg. No. 61842-18	NovaSource, Tessenderlo Kerley, Inc. Phoenix, AZ, USA	60g/1 L water + 10.6 mL Attach® adjuvant (spreader-sticker)
Pyrethrin	Prentox Pyronyl^TM^ Crop Spray EPA Reg. No. 655-489	Prentiss Incorporated LLC, Sandersville, GA, USA	2.64 mL/1 L water
Water (control)			

**Table 4:** Median Chinese rose beetle damage rating of cacao leaves on trees exposed to pest management products or a water-treated control.

Active Ingredient/Treatment	Application	N	Median
Water (control)	Foliar spray	18	6.5
Kaolin clay	Foliar spray	19	4
Imidacloprid	Soil drench	20	3.5
Pyrethrin	Foliar spray	19	3
Imidacloprid	Foliar spray	20	2
*B. bassiana*	Foliar spray	18	1.75
Azadirachtin	Foliar spray	18	0.5

**Table 5:** Longevity of adult Chinese rose beetle when exposed to cacao leaves with different insecticides. Means followed by different letters indicate a statistically significant difference as determined by pairwise comparisons using the Tukey test with α = 0.05.

Active Ingredient/Treatment	N	Mean ± SEM (days)	Min–Max (days)
Water (control)	11	18.91 ± 2.65 ^a^	10–32
*B. bassiana*	10	17.30 ± 2.19 ^ab^	9–32
Kaolin clay	11	11.55 ± 2.06 ^ab^	5–30
Azadirachtin	11	11.45 ± 1.29 ^ab^	6–16
Imidacloprid	14	10.36 ± 1.14 ^b^	5–16
Pyrethrin	14	0 ± 0	0
